# Response of Oral and Skin Keratinocytes to Oxidative Stress

**DOI:** 10.3390/cells15020097

**Published:** 2026-01-06

**Authors:** Yixuan Zhang, Chen Han, Heidi Yuan, Luisa A. DiPietro, Lin Chen

**Affiliations:** 1 Department of Dermatology, The Fourth Affiliated Hospital of Soochow University (Medical Center of Soochow University), Suzhou 215000, China; yixuan92@uic.edu; 2 Center for Wound Healing and Tissue Regeneration, University of Illinois Chicago, Chicago, IL 60612, USA; chan206@uic.edu (C.H.); hyuan22@uic.edu (H.Y.); ldipiet@uic.edu (L.A.D.); 3 Department of Integrative and Translation Physiology, University of Illinois Chicago, Chicago, IL 60612, USA

**Keywords:** oxidative stress, keratinocytes, skin, oral, reactive oxygen species, antioxidant defense

## Abstract

Oxidative stress caused by excessive reactive oxygen species (ROS) disrupts skin and oral epithelial homeostasis and contributes to skin aging, inflammation, periodontitis, and mucosal injury. As the principal defenders in both skin and oral mucosal tissues, keratinocytes are important responders to oxidative stress. However, most existing studies have examined skin or oral keratinocytes in isolation, with few comparative investigations of their tolerance, repair capacity, and antioxidant mechanisms under oxidative stress. In this study, we systematically compared immortalized oral keratinocytes (TIGK) and skin keratinocytes (HaCaT) under hydrogen peroxide (H_2_O_2_)-induced oxidative stress. Functional analyses, including cell survival, ROS accumulation, stress granule formation, in vitro wound healing, and proliferation recovery assays, were combined with transcriptomic profiling to evaluate differences in antioxidant and pro-oxidant systems. TIGK exhibited significantly higher survival rates, lower ROS accumulation, and superior migratory and proliferative recovery compared with HaCaT after oxidative insult. Transcriptomic analysis further revealed that TIGK consistently expressed higher levels of antioxidant genes and enzymes. In contrast, HaCaT showed greater ROS accumulation and relatively limited antioxidant defenses. The results show that oral and skin keratinocytes adopt distinct adaptive mechanisms under oxidative stress. The intrinsic redox advantage of oral keratinocytes provides new insights into their rapid wound-healing capacity and may inform strategies to enhance epithelial resilience.

## 1. Introduction

Response to oxidative stress is a central factor in maintaining epithelial health and tissue homeostasis. Excessive production of ROS beyond the antioxidant capacity of cells damages macromolecules, including DNA, proteins, and lipids, leading to dysfunction, inflammation, senescence, and even apoptosis [1,2,3,4,5]. In the skin, oxidative insults accelerate both intrinsic and extrinsic aging, aggravate chronic inflammation, increase carcinogenic potential, and weaken barrier function [6,7]. Similarly, oral epithelia are exposed to comparable oxidative challenges, as periodontitis and mucosal lesions such as ulcers and mucositis are closely linked to oxidative stress, resulting in epithelial disruption and delayed wound healing [2,8,9]. Keratinocytes are the fundamental cellular units of both skin and oral epithelia, forming the first line of defense against external insults and safeguarding barrier integrity [10,11]. Although they share similar protective roles, differences in local environment, phenotypic features, and functional demands require distinct characteristics and inflammatory adaptability at oral and skin sites [12,13]. Consequently, keratinocyte antioxidant responses to exogenous oxidative stress may exhibit site-specific divergence.

Recent studies have shown that skin keratinocytes rely on multilayered antioxidant defenses under oxidative stress. Enzymatic systems such as superoxide dismutase (SOD), catalase (CAT), and glutathione peroxidase (GPX), together with nonenzymatic antioxidants including glutathione (GSH), vitamin C, and vitamin E, act cooperatively to eliminate excess ROS and maintain redox homeostasis [14]. By contrast, the antioxidant responses of oral keratinocytes remain less well characterized, with only scattered reports suggesting antioxidant activation under conditions such as mucositis or exposure to tooth bleaching reagents [8,15]. However, most existing studies have focused separately on the oxidative stress responses of either skin or oral keratinocytes, with few comparative investigations of their tolerance, repair capacity, and antioxidant mechanisms under oxidative challenge.

This study provides the first systematic comparison between TIGK and HaCaT under H_2_O_2_-induced oxidative stress, encompassing functional parameters including cell survival, ROS accumulation, migratory repair, proliferative recovery, and so on, alongside transcriptomic profiling of antioxidant and pro-oxidant systems. TIGK displayed superior tolerance, repair capacity, and antioxidant enzyme activity compared with HaCaT, and exhibited an inherently more substantial antioxidant potential at the transcriptional level. By contrast, HaCaT showed increased ROS accumulation and relatively constrained antioxidant defenses. These findings reveal distinct adaptive mechanisms of oral and skin keratinocytes under oxidative stress and provide new cellular and molecular insights into their differential resistance to oxidative injury.

## 2. Materials and Methods

### 2.1. Cell Culture

The HaCaT cell line was obtained from AddexBio (San Diego, CA, USA), and the TIGK cell line was obtained from ATCC (Manassas, VA, USA). Both cell types were maintained in DermaLife K Keratinocyte Complete Medium (Lifeline Cell Technology, Frederick, MD, USA), supplemented with TGF-α (0.5 ng/mL), insulin (5 µg/mL), epinephrine (1 µM), L-glutamine (6 mM), pituitary extract (0.4%), apo-transferrin (5 µg/mL), D-glucose (6 mM), and hydrocortisone hemisuccinate (100 ng/mL). Cells were incubated at 37 °C in a humidified atmosphere containing 5% CO_2_. For the corresponding experiments, cells were seeded at densities of 4 × 10^5^ cells per well in 6-well plates, 2 × 10^5^ cells per well in 12-well plates, 3 × 10^4^ cells per well in 48-well plates or 1.5 × 10^4^ cells per well in 96-well plates, and cultured for use on the following day, unless otherwise stated.

### 2.2. Cell Viability and Proliferation Assay

For the cell viability assay, single-cell suspensions were prepared by detaching adherent cells from 12-well plates using TrypLE (Gibco, Waltham, MA, USA). Trypan blue was added to the cell suspension, and viable cells were quantified using a TC20 Automated Cell Counter (Bio-Rad, Hercules, CA, USA). Cells without H_2_O_2_ treatment served as the control group. N-acetylcysteine (NAC, 15 mM) (Sigma-Aldrich, St. Louis, MO, USA) was included to confirm that the observed cellular damage was specifically induced by H_2_O_2_ and to assess the recovery capacity of the two cell types following H_2_O_2_ exposure.

For the cell proliferation/cytotoxicity assay, cells were seeded into 96-well plates and treated with the corresponding concentrations of H_2_O_2_ in the medium. After treatment, 10 µL of MTS reagent (Abcam, Cambridge, UK) was added to each well containing 100 µL of culture medium, and cells were incubated at 37 °C for 2 h according to the manufacturer’s instructions. The absorbance at 490 nm was then measured using the SpectraMax Plus microplate reader (Molecular Devices, Sunnyvale, CA, USA). Results were normalized to the control group by dividing the optical density (OD) of treated samples by the OD of the control. For the detection of recovery proliferation, after one day of recovery following H_2_O_2_ treatment, cells were dissociated, reseeded in a 96-well plate, and then subjected to the MTS assay.

### 2.3. Assessment of Cellular Apoptosis

Apoptosis was evaluated using Annexin V staining. TIGK and HaCaT were seeded into 6-well plates and treated with the corresponding concentrations of H_2_O_2_ in serum-free medium for 3 h. Following treatment, cells were stained with Annexin V-FITC according to the manufacturer’s instructions of Annexin V-FITC Apoptosis Detection Kit (Biotium, Fremont, CA, USA). Briefly, without removing the culture medium, 1/10 volume of 10× Annexin V solution (final concentration: 0.25 µg/mL) was added directly to each well and mixed gently to avoid uneven staining. Cells were incubated in the dark at 37 °C for 30 min prior to imaging.

Fluorescence images were acquired using an inverted fluorescence microscope (Carl Zeiss Microscopy GmbH, Göttingen, Germany). For each cell type and time point, three replicate wells were analyzed. In each well, 3–5 random fields were imaged, and Annexin V-positive cells were manually counted. The apoptosis rate was determined by calculating the percentage of Annexin V-positive cells relative to the total number of cells in each field, and the mean value for each well was used for statistical analysis.

### 2.4. Detection of Intracellular Reactive Oxygen Species

Intracellular ROS levels were measured using CM-H_2_DCFDA (Thermo Fisher Scientific, Waltham, MA, USA). TIGK and HaCaT were seeded in 96-well black-wall plates and allowed to adhere overnight. The medium was then replaced, and cells were cultured under standard conditions until reaching 80% confluency. For ROS staining, cells were pre-incubated with 5 µM CM-H_2_DCFDA in the dark at 37 °C for 30 min in a humidified incubator. The cells were then treated with 5 mM H_2_O_2_, with or without NAC for 3 h at 37 °C. Fluorescence intensity was measured using the Synergy microplate reader (BioTek, Winooski, VT, USA) at an excitation/emission wavelength of 485/528 nm. In addition, stained cells were visualized and imaged using the inverted fluorescence microscope. Relative ROS levels were normalized to the control group (cells without H_2_O_2_ treatment).

### 2.5. Immunofluorescence Staining for Stress Granules (SG)

TIGK and HaCaT cells were seeded in 8-well chamber slides and cultured under standard conditions until reaching approximately 60–70% confluency. Cells were fixed with 4% paraformaldehyde (Electron Microscopy Sciences, Hatfield, PA, USA) for 15 min at room temperature and washed three times with 1× PBS. Permeabilization was performed with 0.15% Triton X-100 in PBS for 10 min, followed by blocking with 5% goat serum in PBS for 1 h at room temperature. Cells were incubated overnight at 4 °C with mouse anti-human G3BP1 antibody (Proteintech, 66486-1-Ig, Rosemont, IL, USA; 1:300). After washing with PBS, cells were incubated for 1 h at room temperature in the dark with Alexa Fluor 488-conjugated goat anti-mouse IgG (Invitrogen, Waltham, MA, USA; 1:1000). Nuclei were counterstained with DAPI (Thermo Fisher Scientific, Waltham, MA, USA). Fluorescence images were acquired using the Axioskop 40 fluorescent microscope (Carl Zeiss Microscopy GmbH). Each treatment group included three biological replicates.

For each well, images of three randomly selected fields were captured using a 40× objective. The percentage of SG-forming cells was calculated as the number of SG-positive cells divided by the total number of cells (DAPI-positive cells) in the field.

### 2.6. Hydrogen Peroxide Assay

TIGK and HaCaT were seeded into 6-well plates and cultured under the same conditions as described for the viability assay. Five mM H_2_O_2_ was added to the culture medium, and cells were incubated at 37 °C. At the indicated time points, 200 µL of culture supernatant was collected. To deproteinize the samples, a 10 kDa molecular weight cut-off spin column (MilliporeSigma, Burlington, MA, USA) was used by centrifugation at 14,000× *g* for 10 min. The concentration of H_2_O_2_ in the culture supernatant was determined using a Hydrogen Peroxide Assay Kit (Abcam, Cambridge, UK) following the manufacturer’s instructions. Fluorescence intensity was measured on the Synergy microplate reader (BioTek, Winooski, VT, USA) at an excitation/emission wavelength of 535/587 nm. Dilution factors (1/10 for wells with cells and 1/50 for wells without cells) were selected based on preliminary calibration to ensure that all values fell within the linear range of the standard curve.

### 2.7. Cell Migration Assay—In Vitro Wound Healing

Cell migration was assessed using an in vitro wound-healing assay. TIGK and HaCaT were seeded into 12-well plates and cultured until reaching confluency. Cells were pretreated with mitomycin-C (1 μg/mL, Sigma-Aldrich) for 1 h at 37 °C to inhibit proliferation prior to scratching. Cells were then treated with 5 mM H_2_O_2_ in the medium for 30 min at 37 °C. Following treatment, a linear scratch was made in each well using a sterile 200 µL pipette tip to create a wound across the cell monolayer. After scratching, wells were gently washed twice with PBS (Gibco, Waltham, MA, USA) to remove detached cells, and fresh growth medium was added. Images of the wound area were captured at 0, 24, 48, and 72 h after scratching. Images were captured at the same predefined position in each well to ensure consistent tracking of the wound area. The open wound areas were quantified using ImageJ software (v1.48v). The migration rate was calculated as the percentage of wound closure at each time point relative to the initial wound area.

### 2.8. Detection of Antioxidants

HaCaT and TIGK were cultured until reaching 90% confluency in 12-well plates. Cells were then treated with or without 5 mM H_2_O_2_ for 3 h. Cells collected after treatment underwent three cycles of freezing and thawing in cold PBS containing the protease inhibitor phenylmethylsulfonyl fluoride (1 mM; Sigma-Aldrich, St. Louis, MO, USA), followed by sonication. The supernatants were obtained from the cell lysates by centrifugation at 4 °C and 16,000× *g* for 15 min. Total protein concentration of the lysates was determined using the Pierce™ BCA Protein Assay Kit (Thermo Fisher Scientific, Waltham, MA, USA) for normalization. The protein levels of Superoxide Dismutase 1 (SOD1), Glutathione S-Transferase Mu 3 (GSTM3), and Lactoperoxidase (LPO) were quantified using the Human SOD1 ELISA Kit (Abcam, Cambridge, UK), Human GSTM3 ELISA Kit (Thermo Fisher Scientific, Waltham, MA, USA), and Human LPO ELISA Kit (Biorbyt, Cambridge, UK), respectively, following the manufacturer’s protocols. Protein levels were calculated from the respective standard curves and then normalized to total protein according to the formula: C_Normalized protein_ = C_Target protein_/C_Total protein_.

In addition, Thioredoxin Reductase (TrxR) activity was measured using the TrxR Microplate Assay Kit (Biorbyt, Cambridge, UK) according to the manufacturer’s instructions. In the assay, TrxR catalyzes the reduction of 5, 5′-dithiobis (2-nitrobenzoic) acid (DTNB) with NADPH to 5-thio-2-nitrobenzoic acid (TNB2-). One unit of TrxR activity is defined as the amount of enzyme which produces 1 μmol of TNB per minute at 37 °C, which was calculated according to the formula provided in the manual: TrxR (U/mg) = 0.5 × (OD_Standard_ − OD_Blank_)/(OD_Sample_ − OD_Control_)/C_Total protein_.

According to the manufacturers, these kits are specifically designed for the quantification of each target and exhibit no significant cross-reactivity with related analogs.

### 2.9. Transcriptome Sequencing of Baseline and H_2_O_2_-Treated HaCaT and TIGK

Total RNA was extracted from HaCaT and TIGK in 6-well plates treated with 5 mM H_2_O_2_ for 3 h or left untreated (*n* = 3 per group) using TRIzol (Invitrogen, Waltham, MA, USA), purified with the RNA Clean and Concentrator-25 kit (Zymo, Tustin, CA, USA), and treated with DNase I (Thermo Fisher Scientific, Waltham, MA, USA). RNA integrity was assessed with the Agilent 2100 Bioanalyzer, and all samples had RNA integrity number values above 8.9. RNA-seq generated libraries were checked with Qubit and real-time (RT) PCR for quantification and bioanalyzer for size distribution detection. Quantified libraries were then sequenced on the Illumina platform by Novogene (Novogene America, Sacramento, CA, USA) using in-house perl scripts. Clean reads were mapped to the human reference genome (GRCh38/hg38) using Hisat2 (v2.0.5), and the mapped reads were assembled by StringTie (v1.3.3b) [16]. Raw counts of read numbers mapped to each gene were generated in R using the FeatureCounts package (v1.5.0-p3) [17]. Library preparation, genome mapping, and raw gene counts for mRNA-sequencing analysis were performed by Novogene (Novogene America). Correlation heatmaps were generated utilizing raw counts for each mapped gene.

Differentially expressed gene (DEG) analysis of the raw count data was performed using DESeq2 (v1.20.0) in R (v4.4.1) with Bioconductor v3.20 [18]. Genes with an average raw count ≥ 10 across all samples were included, and the false discovery rate (FDR) was controlled by Benjamini–Hochberg correction [18,19]. Genes with an adjusted *p* (*p*.adj) < 0.05 following FDR correction were assigned as being differentially expressed. Furthermore, raw count data underwent variance-stabilized transformation using the vst() function in DESeq2 for principal component analysis (PCA), which was visualized using each sample’s top 500 highly variable genes [18]. Normalized count values for the DEGs in each sample were also retrieved as part of our DESeq2 analysis for each of our comparisons [18]. This was performed by using the counts() function and adding the argument normalized = TRUE [18].

In this study, we assessed differentially regulated genes in HaCaT versus TIGK at baseline and after H_2_O_2_-treatment using the following parameters: *p*.adj < 0.01 and log (fold change) > 0 or log (foldchange) < 0. Genes with a negative log (foldchange) were considered upregulated in TIGK, while genes with a positive log(fold change) were considered upregulated in HaCaT. Differentially regulated genes in HaCaT or TIGK following H_2_O_2_-treatment relative to their own respective baseline were assessed by using the following parameters: *p*.adj < 0.01 and log (fold change) > 0 or log (foldchange) < 0. Genes with a negative log (fold change) were considered downregulated in HaCaT or TIGK following H_2_O_2_-treatment, while genes with a positive log (fold change) were considered upregulated in HaCaT or TIGK following H_2_O_2_-treatment. The gene sets for antioxidants and pro-oxidants were first obtained from the GeneCards database (https://www.genecards.org/) and then curated based on previous literature [20,21,22], and are provided in Supplementary Tables S1 and S2. Z-scores for antioxidants and pro-oxidant genes were generated using normalized gene counts and plotted as a heatmap in R [23]. Venn diagrams were made with Venny [24].

GO BP and Reactome pathway term enrichment analysis was performed on the list of differentially upregulated and downregulated genes in HaCaT relative to TIGK at baseline and after H_2_O_2_-treatment via the EnrichR package in R version 4.4.1 [25,26,27]. Enrichment was also performed on the list of differentially upregulated and downregulated genes in HaCaT or TIGK following H_2_O_2_-treatment relative to their own respective baseline. GO terms with a *p*.adj < 0.05 following Benjamini and Hochberg’s approach for controlling the FDR were considered statistically significantly enriched. Up to 10 significantly enriched terms based on *p*.adj were utilized for visualization.

### 2.10. Statistical Analysis

All data are expressed as mean ± standard deviation (SD). Normality was assessed using the Shapiro–Wilk test. Statistical comparisons were performed using two-way ANOVA followed by Benjamini, Krieger, and Yekutieli post hoc test or Two-tailed unpaired *t*-test using GraphPad Prism 10.0 (GraphPad, San Diego, CA, USA). When *p* < 0.05 it was considered statistically significant.

## 3. Results

### 3.1. TIGK Exhibits Higher Tolerance to H_2_O_2_-Induced Oxidative Injury

**Figure 1:**
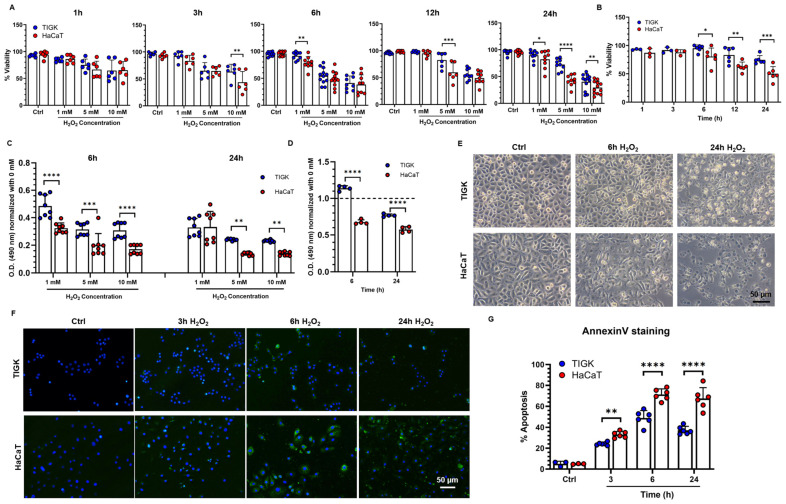
Oral keratinocytes are more tolerant to H_2_O_2_-induced oxidative injury than skin keratinocytes. (**A**) Viability of oral keratinocytes (TIGK) and skin keratinocytes (HaCaT) under different concentrations of H_2_O_2_ and at different time points. The data shown is pooled from 3–4 repeated experiments. (**B**) Antioxidant effect of NAC in TIGK and HaCaT after 5 mM H_2_O_2_ treatment, as assessed by viability assay. (**C**) Cytotoxicity of different H_2_O_2_ concentrations in TIGK and HaCaT, as assessed by MTS assay. The data shown are representative of 3 experiments. (**D**) Antioxidant effect of NAC in TIGK and HaCaT after 5 mM H_2_O_2_ treatment, as assessed by MTS assay. The dotted line indicates the level of the untreated control. (**E**) Representative bright-field images of TIGK and HaCaT treated with 5 mM H_2_O_2_. (**F**) Apoptosis was assessed by Annexin V staining. TIGK and HaCaT were treated with 5 mM H_2_O_2_ for 3, 6, and 24 h. Representative images of Annexin V staining. Blue indicates DAPI nuclear staining, and green indicates Annexin V–positive cells. (**G**) Quantification of apoptosis assessment. The data shown is pooled from 3 experiments. Data are shown as mean ± SD. * *p* < 0.05, ** *p* < 0.01, *** *p* < 0.001, **** *p* < 0.0001. Two-way ANOVA followed by the Benjamini, Krieger, and Yekutieli post hoc test was used for (**A**–**D**,**G**).

To compare the tolerance of oral and skin keratinocytes to oxidative stress, TIGK and HaCaT were exposed to various concentrations of H_2_O_2_, and cell viability was assessed at different time points (Figure 1A; Supplementary Figure S1A). After 1 h and 3 h of exposure, no significant differences in viability were observed between the two cell types, except that TIGK exhibited higher viability than HaCaT under 10 mM H_2_O_2_ at 3 h. At 6, 12, and 24 h, the effect of H_2_O_2_ on cell viability became more pronounced. Under 5 mM H_2_O_2_, TIGK consistently maintained significantly higher viability than HaCaT at 12 and 24 h (Figure 1A). Adding the antioxidant NAC (15 mM) markedly improved the viability of both cell types, with a more pronounced recovery observed in TIGK than in HaCaT (Figure 1B). In addition, MTS assays showed H_2_O_2_ was less cytotoxic to TIGK than to HACAT after 6 h and 24 h of exposure (Figure 1C), and NAC treatment markedly reduced H_2_O_2_-induced cytotoxicity in both cell types, with TIGK showing an even greater recovery of metabolic activity, exceeding control levels (Figure 1D). Continuous monitoring of cell viability following H_2_O_2_ treatment revealed that at a concentration of 5 mM, the viability difference between the two cell types progressively increased in a time-dependent manner (Supplementary Figure S1A), indicating that 5 mM H_2_O_2_ was optimal for inducing marked differential responses, so this concentration was selected for subsequent experiments. In addition, we measured the degradation of H_2_O_2_ and found that, in the presence of cells, H_2_O_2_ was completely degraded within approximately 3 h (Supplementary Figure S1B). Both TIGK and HaCaT displayed irregular cellular morphology accompanied by nuclear pyknosis and cell death following H_2_O_2_ treatment, but this phenotype was more pronounced in HaCaT (Figure 1C). To assess the impact of oxidative stress on apoptosis, Annexin V staining was performed after 3, 6, and 24 h of H_2_O_2_ exposure (Figure 1D,E). At all time points examined, the proportion of apoptotic cells was significantly higher in HaCaT than in TIGK (Figure 1E).

### 3.2. TIGK Accumulates Lower Levels of ROS and Lower Stress Granule Formation Following H_2_O_2_ Treatment

**Figure 2:**
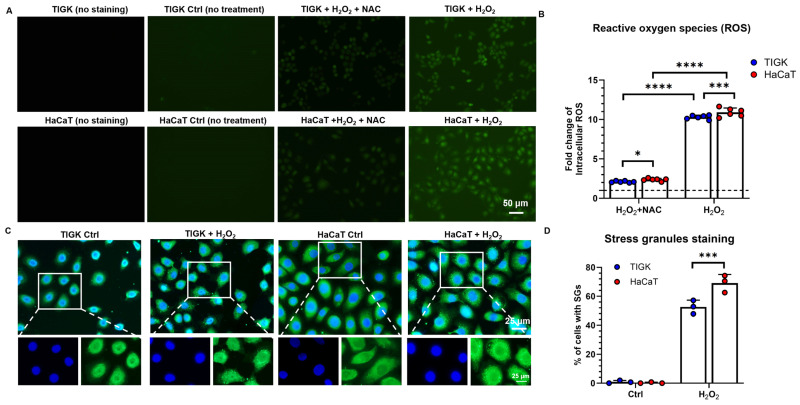
Oral keratinocytes and skin keratinocytes exhibit different oxidative stress responses following H_2_O_2_-induced injury. (**A**) Representative fluorescence images showing ROS levels (green) in TIGK and HaCaT after 5 mM H_2_O_2_ or H_2_O_2_ + NAC treatment for 3 h. (**B**) Quantification of ROS levels normalized to untreated control. The dotted line represents the level of the untreated control. (**C**) Representative fluorescence images showing stress granule (SG) formation in TIGK and HaCaT cells before and after treatment with 500 μM H_2_O_2_ for 2 h. Cells were stained with G3BP1 (green) for SG detection and DAPI (blue) for nuclei counterstaining. (**D**) Quantification of the percentage of cells containing SG in TIGK and HaCaT under control and H_2_O_2_-treated conditions. Data are shown as mean ± SD. * *p* < 0.05, *** *p* < 0.001, **** *p* < 0.0001. Two-way ANOVA followed by the Benjamini, Krieger, and Yekutieli post hoc test was used for (**B**,**D**).

To compare the generation of ROS between oral and skin keratinocytes under oxidative stress, intracellular ROS levels were measured in TIGK and HaCaT after 3 h of H_2_O_2_ exposure (Figure 2A,B). Compared with untreated controls, H_2_O_2_ treatment markedly increased ROS accumulation in both cell types (Figure 2A), with HaCaT exhibiting significantly higher ROS levels than TIGK. Adding NAC substantially reduced ROS levels in both cell types, and under NAC treatment, ROS levels in TIGK remained significantly lower than those in HaCaT (Figure 2B). Skin keratinocytes form SG under oxidative stress [28]. To further investigate the cellular stress response under oxidative conditions, we examined SG formation following treatment with H_2_O_2_. In untreated control cells, almost no SGs were visible. However, when the cells were treated with 500 μM H_2_O_2_ for 2 h, a significant increase in SG formation was observed in both HaCaT and TIGK, particularly in HaCaT cells (Figure 2C). Quantitative analysis indicated that 50% of TIGK cells formed SGs, compared to 70% in HaCaT cells (*p* < 0.001, Figure 2D) following H_2_O_2_ treatment.

### 3.3. Transcriptomic Differences Between TIGK and HaCaT at Baseline and After H_2_O_2_ Treatment

**Figure 3:**
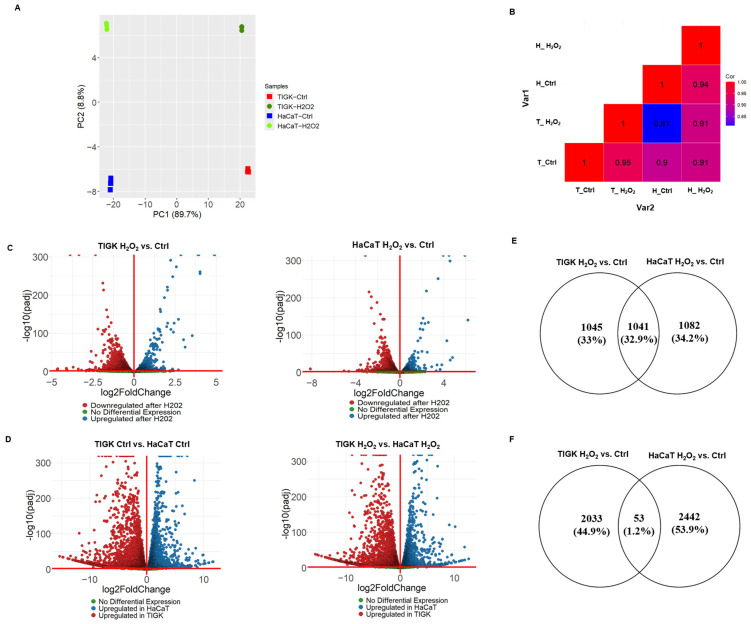
Oral and skin keratinocytes show distinct transcriptional responses to H_2_O_2_-induced oxidative injury. (**A**) PCA of mRNA-sequencing data from TIGK and HaCaT with or without H_2_O_2_ treatment. Each colored point represents an individual sample. The x-axis and y-axis indicate the first and second principal components. (**B**) Heatmap showing Pearson correlation coefficients between experimental (groups TIGK and HaCaT with or without H_2_O_2_). Each square represents the average correlation between gene expression profiles from the indicated groups, highlighting both cell type and treatment-specific transcriptomic differences. (**C**) Volcano plots showing DEGs in HaCaT and TIGK after H_2_O_2_ treatment compared to their respective controls. (**D**) Volcano plots comparing DEGs between HaCaT and TIGK under H_2_O_2_ treatment or control conditions. (**E**) Venn diagram comparing the genes upregulated by H_2_O_2_ in HaCaT and TIGK. (**F**) Venn diagram comparing the genes downregulated by H_2_O_2_ in HaCaT and TIGK.

To further understand the observed differences between oral and skin keratinocytes under oxidative stress, we performed mRNA sequencing of TIGK and HaCaT under H_2_O_2_-treated and control conditions. PCA revealed that the first two principal components accounted for 89.7% and 8.8% of the variance, respectively, with samples clustering primarily by cell type and secondarily by treatment status (H_2_O_2_ vs. control) (Figure 3A). Pearson correlation heatmap analysis further demonstrated that gene expression profiles within the same cell type across treatments were more highly correlated than those between different cell types, indicating that transcriptomic differences were predominantly determined by cell identity, while H_2_O_2_ treatment elicited marked expression changes within each cell type (Figure 3B).

Differential expression analysis revealed a large number of DEGs in both TIGK and HaCaT after H_2_O_2_ treatment compared to their respective controls (Figure 3C). There were 2086 genes upregulated in TIGK and 2123 genes upregulated in HaCaT, with 1041 genes shared between the two cell types (Figure 3E). Downregulated gene analysis revealed 2086 genes downregulated in TIGK and 2495 genes downregulated in HaCaT, with only 53 shared genes downregulated in both cell types (Figure 3F). TIGK and HaCaT showed cell type-specific differential expression patterns for many genes before and after H_2_O_2_ treatment (Figure 3D). Upregulated genes in TIGK were enriched in GO terms related to transcriptional regulation, stress response, and cell cycle regulation, whereas upregulated genes in HaCaT were associated with terms such as steroid biosynthetic process, cellular response to stress, and signaling by the Rho GTPase-mediated pathway (Supplementary Figures S2 and S3). Both HaCaT and TIGK exhibited marked transcriptomic differences under baseline and oxidative stress conditions, with H_2_O_2_-induced changes showing pronounced cell–type–specific expression patterns, which may be closely related to their distinct tolerance to oxidative injury.

### 3.4. TIGK and HaCaT Exhibit Distinct Expression Profiles of Antioxidant Genes

**Figure 4:**
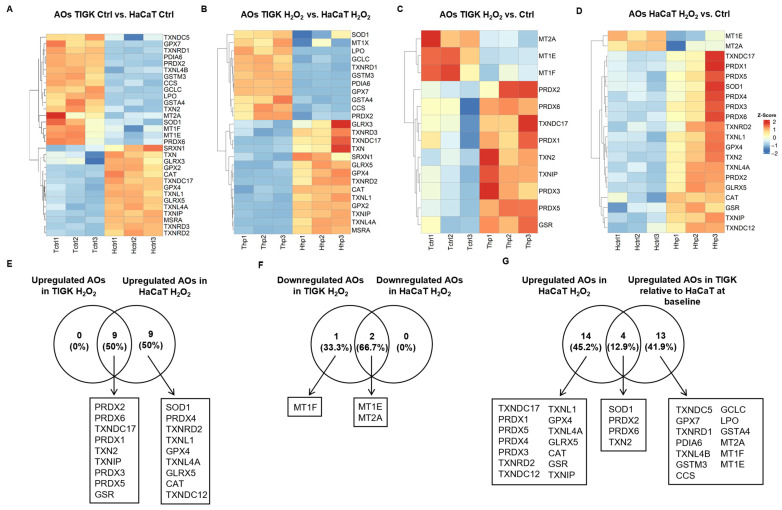
Oral and skin keratinocytes exhibit significant differences in their expression of antioxidant genes (AOs) at baseline and following H_2_O_2_-induced oxidative injury. (**A**–**D**) Heatmaps showing differentially expressed AOs between HaCaT and TIGK under basal conditions, after H_2_O_2_ treatment, and relative to respective controls. Each column represents an individual sample, and each row an AO gene. (**E**–**G**) Venn diagrams comparing the shared numbers of upregulated and downregulated AOs in HaCaT and TIGK after H_2_O_2_ treatment, as well as the shared upregulated AOs in HaCaT and TIGK relative to HaCaT at baseline after H_2_O_2_ treatment.

Antioxidant genes (AOs) play a crucial role in maintaining cellular redox homeostasis and defending against oxidative stress. We compared the AO expression profiles of TIGK and HaCaT under baseline conditions (control) and following H_2_O_2_ treatment. Under baseline conditions, TIGK exhibited higher expression of multiple AOs, such as *LPO*, *GPX7*, and *SOD1*, whereas HaCaT showed higher levels of other AOs, such as *GLRX3*, *GPX2*, and *CAT* (Figure 4A). Following H_2_O_2_ treatment, the two cell types continued to display distinct AO expression patterns (Figure 4B). Further comparison of changes in the two cell types after H_2_O_2_ treatment relative to their respective baselines (Figure 4C,D) revealed that all AOs upregulated in TIGK overlapped with those in HaCaT, yet HaCaT expressed another 9 unique AOs (Figure 4E). Enrichment analysis revealed that most of the AOs upregulated in HaCaT were associated with ROS response and protein repair, while the AOs upregulated in TIGK, apart from cellular response to stress, were more skewed toward metal ion metabolism (Supplementary Figures S4 and S5). In the downregulated AO set, TIGK shared 2 genes with HaCaT (Figure 4F). Notably, 4 of the 18 AOs upregulated in HaCaT after H_2_O_2_ exposure (*SOD1*, *PRDX2*, *PRDX6*, and *TXN2*) were already highly expressed in TIGK at baseline relative to HaCaT, representing 22.2% of the HaCaT upregulated genes (Figure 4G, Supplementary Table S3). In summary, TIGK and HaCaT display pronounced differences in AO expression both at baseline and following H_2_O_2_ treatment, and some AOs with high baseline expression in TIGK may play a key protective role in oxidative stress.

### 3.5. TIGK and HaCaT Exhibit Distinct Expression Profiles of Pro-Oxidant Genes

**Figure 5:**
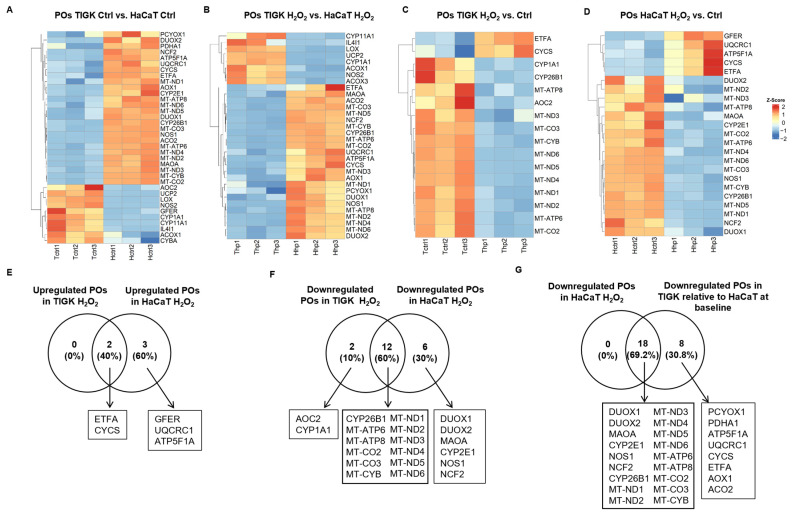
Oral and skin keratinocytes exhibit significant differences in their expression of pro-oxidant genes (POs) at baseline and following H_2_O_2_-induced oxidative injury. (**A**–**D**) Heatmaps showing differentially expressed POs between HaCaT and TIGK under basal conditions, after H_2_O_2_ treatment, and relative to respective controls. Each column represents an individual sample, and each row a PO gene. (**E**–**G**) Venn diagram comparing the shared upregulated and downregulated POs in HaCaT and TIGK after H_2_O_2_ treatment, as well as the shared downregulated POs in HaCaT and TIGK relative to HaCaT at baseline after H_2_O_2_ treatment.

Pro-oxidant genes (POs) play important roles in regulating intracellular redox balance, generating ROS, and mediating signal transduction. We compared the expression profiles of POs in TIGK and HaCaT under both baseline and H_2_O_2_-treated conditions. At baseline, compared with HaCaT, TIGK had fewer upregulated POs, whereas HaCaT showed higher expressions of more POs, such as *DUOX2*, *NOS1*, and *MAOA* than TIGK (Figure 5A). Following H_2_O_2_ treatment, TIGK still exhibited fewer POs (Figure 5B). Moreover, the upregulated genes in TIGK were more enriched in PPAR signaling and peroxisomal functions, whereas the upregulated POs in HaCaT were primarily associated with energy metabolism and ROS generation (Supplementary Figures S6 and S7). Comparison of the post-H_2_O_2_ profiles relative to their respective baselines (Figure 5C,D) revealed that the 2 upregulated POs in TIGK were shared with HaCaT (*ETFA*, *CYCS*), and HaCaT exhibited another 3 specific AOs (*GFER*, *UQCRC1*, *ATF5F1A*) (Figure 5E). For downregulated POs, 12 were shared between HaCaT and TIGK (Figure 5F). Interestingly, all 18 POs downregulated in HaCaT after H_2_O_2_ treatment were already expressed at lower levels in TIGK under baseline conditions (Figure 5G, Supplementary Table S4). Together, these results indicate that HaCaT and TIGK exhibit marked differences in PO expression both at baseline and after oxidative challenge, and that the inherently lower baseline expression of certain POs in TIGK may help maintain reduced ROS production under oxidative stress.

### 3.6. TIGK and HaCaT Exhibit Distinct Antioxidant and Recovery Abilities Following H_2_O_2_-Induced Oxidative Injury

**Figure 6:**
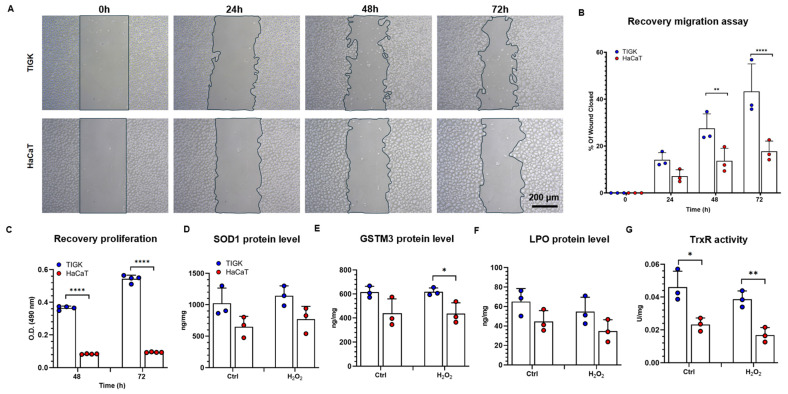
Oral and skin keratinocytes exhibit different antioxidant and recovery abilities following H_2_O_2_-induced oxidative injury. (**A**) The Recovery migration abilities of TIGK and HaCaT were assessed by the scratch assay after 5 mM H_2_O_2_ treatment for 30 min. Representative images of migration are shown. (**B**) Quantification of wound closure. (**C**) Recovery proliferation of TIGK and HaCaT was assessed by MTS assay after 5 mM H_2_O_2_ treatment for 30 min. (**D**–**F**) Protein levels of SOD1, GSTM3, and LPO were measured by ELISA, expressed as ng/mg of total protein. (**G**) TrxR assay is presented as TrxR activity in U/mg. Data are shown as mean ± SD. * *p* < 0.05, ** *p* < 0.01, **** *p* < 0.0001. Two-way ANOVA followed by the Benjamini, Krieger, and Yekutieli post hoc test was used for (**B**,**C**). Two-tailed unpaired *t*-test was used for (**D**–**G**).

To validate the antioxidant differences observed in transcriptomic analyses, we functionally assessed the recovery capacity of TIGK and HaCaT after H_2_O_2_-induced oxidative stress. Scratch assays revealed that after 30 min exposure to 5 mM H_2_O_2_, TIGK displayed significantly higher wound closure rates than HaCaT at both 48 h and 72 h (Figure 6A,B), indicating superior migratory repair ability in TIGK. Consistently, MTS proliferation assays demonstrated that TIGK exhibited significantly greater proliferative recovery at 48 h and 72 h compared to HaCaT (Figure 6C), suggesting enhanced cellular viability in TIGK under oxidative challenge. To further probe their intrinsic antioxidant capacities, we measured the protein levels of SOD1, LPO, and GSTM3, as well as TrxR activity. ELISA results showed elevated levels of SOD1, LPO, and GSTM3 in TIGK compared to HaCaT (Figure 6D–F), both at baseline and after H_2_O_2_ treatment, which were consistent with the gene expression analysis findings. Similarly, TIGK exhibited significantly higher TrxR activity than HaCaT (Figure 6G). Together, these results demonstrate that TIGK possesses superior antioxidant defenses and recovery capacity, including enhanced migration, proliferation, and antioxidant enzymatic activity, following oxidative injury.

## 4. Discussion

Both the skin and oral mucosa contain epithelial barriers composed of keratinocytes, yet they exhibit distinct physiological functions and repair capacities: oral mucosal wounds heal faster, display reduced inflammatory responses, and generate markedly less scarring than skin. These features are thought to be closely associated with the unique molecular characteristics and stress response patterns of oral keratinocytes [29,30,31]. Oxidative stress, caused by the excessive accumulation of ROS, leads to cellular injury and is implicated in apoptosis, senescence, inflammation, and tissue repair [3,4,5,32]. It represents a common challenge for skin and oral epithelia upon mechanical injury, inflammation, ultraviolet radiation, or chemical exposure. Previous studies have shown that keratinocytes from both tissues can undergo functional impairment or even cell death under oxidative stress [8,15,33]. However, a systematic comparison of oral and skin keratinocyte responses to oxidative stress is still lacking. Here, we aimed to delineate the differential adaptive mechanisms of these cells under oxidative stress, thereby providing new molecular insights into the distinct healing outcomes of skin and oral tissues. To address this question, we employed H_2_O_2_ to induce oxidative stress. The responses of skin- and oral-derived keratinocytes were compared before and after injury, with transcriptomic profiling integrated to uncover their molecular basis and provide evidence for tissue-specific antioxidant differences. For this purpose, two widely used immortalized keratinocyte lines, HaCaT (skin-derived) and TIGK (oral-derived), were employed, as they have been shown to closely resemble primary keratinocytes in multiple functional aspects and thus represent robust in vitro models for dissecting differential responses between skin and oral keratinocytes [13]. In addition, compared with primary keratinocytes, these cell lines offer advantages of reproducibility, extended lifespan, and reduced inter-individual variability [34,35], and have been extensively applied in studies of skin and oral epithelia in vitro [34,35,36,37].

In this study, we observed that TIGK exhibited significantly higher survival rates than HaCaT under oxidative stress, accompanied by markedly lower intracellular ROS accumulation, stronger recovery capacity, and decreased formation of SGs, which serve as protective structures that help sustain cellular homeostasis under stress [38,39], collectively indicating a greater tolerance of TIGK to oxidative stress injury. This conclusion is consistent with our previous report on temperature-induced stress, in which TIGK also displayed greater thermotolerance following heat exposure compared with HaCaT [35]. We further found that exogenous H_2_O_2_ was degraded shortly (within approximately 3 h) in the presence of cells, consistent with previous reports on H_2_O_2_ dynamics [40]. Nevertheless, differences in survival and apoptosis between the two cell types persisted even after H_2_O_2_ degradation, indicating that oxidative injury may be determined not solely by the presence of exogenous H_2_O_2_ but rather by the intrinsic capacity of cells to scavenge and counteract ROS. Our data suggest that the enhanced tolerance of TIGK to oxidative injury may derive from a more robust endogenous antioxidant defense system.

Our transcriptomic analysis of AOs showed that TIGK and HaCaT displayed distinct AO expression profiles under basal and H_2_O_2_-treated conditions. Even at baseline, TIGK exhibited higher expression of several critical AOs. Notably, among the 18 AOs upregulated in HaCaT after H_2_O_2_ exposure, four genes (*SOD1*, *PRDX2*, *PRDX6*, and *TXN2*) were already highly expressed in TIGK at baseline, all of which play essential roles in maintaining cellular redox homeostasis. *SOD1* is a classical antioxidant enzyme responsible for scavenging superoxide anions [41,42], *PRDX2* and *PRDX6* participate in the elimination of H_2_O_2_ and lipid peroxides [43,44], while *TXN2* regulates mitochondrial redox balance [45]. These findings indicate that part of the antioxidant defense in TIGK was already relatively active before the oxidative challenge. Following H_2_O_2_ exposure, HaCaT upregulated a larger set of AOs than TIGK, suggesting that HaCaT requires broader mobilization of antioxidant responses to cope with oxidative stress. By contrast, the intrinsic advantage of TIGK may enable it to initiate defense mechanisms more rapidly at the early stage of oxidative stress, thereby limiting sustained ROS-induced damage. Comparison of functional enrichment analysis before and after stress revealed that, in addition to the oxidative stress response shared by both cell types, TIGK showed a marked activation of metal ion response pathways. Metal ion homeostasis, particularly of copper, iron, and zinc, is tightly linked to ROS generation and detoxification, and dynamic regulation of these transition metals is considered a key determinant of cellular susceptibility to oxidative damage [46]. The ability of TIGK to mobilize metal ion responses under oxidative stress may therefore provide a molecular basis for its greater tolerance. Furthermore, several antioxidant genes, including *CCS*, *SOD1*, *LPO*, *GSTM3*, *GPX7*, and *TXNRD1*, were consistently higher in TIGK than in HaCaT under basal and stressed conditions, a finding further supported by our antioxidant enzyme assays. These genes represent distinct antioxidant pathways: *CCS* is the copper metallochaperone for *SOD1* and cooperates with *SOD1* in superoxide clearance [42]; *LPO* contributes to hydrogen peroxide utilization and antimicrobial defense [47]; *GSTM3*, *GPX7*, and *TXNRD1* belong to the classical selenium-dependent GPX/TXNRD families that form the core of cellular redox control [41,48]. Together, these multi-pathway and multi-level antioxidant mechanisms enable TIGK to exhibit a more robust antioxidant capacity than HaCaT.

TIGK and HaCat also exhibited marked differences in their relative expression of POs. At baseline, HaCaT upregulated more POs, including classical family members such as *DUOX2* and *NOS1*. *DUOX2*, a member of the NADPH oxidase family, directly catalyzes superoxide production [49], while *NOS* generates nitric oxide that can react to form peroxynitrite, thereby exacerbating oxidative stress [50]. Furthermore, many of the POs downregulated in HaCaT following H_2_O_2_ exposure were already expressed at lower levels in TIGK under basal conditions. This suggests that, compared with HaCaT, TIGK does not rely on extensive suppression of pro-oxidant pathways to cope with exogenous oxidative pressure, but instead benefits from inherently low baseline expression, thereby maintaining a naturally reduced capacity for ROS generation. The pro-oxidant background of HaCaT is more oriented toward rapid ROS generation and mitochondria-related energy metabolism, which may facilitate short-term responses to environmental changes but is also more likely to result in excessive ROS accumulation and cellular damage under sustained stress. In contrast, the pro-oxidant expression profile of TIGK is more consistent with metabolic homeostasis. Although it includes some ROS-generating functions, it predominantly involves peroxisomal pathways that maintain low-level oxidative reactions [51], and its upregulated POs are primarily associated with PPAR signaling, a pathway closely linked to the maintenance of low-level ROS signaling and metabolic adaptation [52,53]. This low pro-oxidant baseline, combined with a metabolism-centered expression pattern, reduces the burden on the antioxidant system and confers a greater advantage to TIGK in withstanding long-term oxidative environments.

By integrating transcriptomic profiling with functional assays, this study uncovered distinct oxidative stress responses between TIGK and HaCaT, although several limitations should be acknowledged. Our analysis primarily focused on mRNA-level changes, whereas oxidative stress responses are also critically regulated at the post-translational level, including phosphorylation, acetylation, and nitration, as well as protein degradation [54]. Although our enrichment analysis indicated biological processes related to “protein repair” and “protein modification”, these findings remain confined to transcriptomic data and cannot fully capture the impact of post-translational modifications in oxidative stress responses. Future integration of proteomics and post-translational modification profiling will be essential to elucidate more comprehensive mechanisms underlying redox regulation in oral versus skin keratinocytes. It is well established that ROS generation and clearance are closely linked to mitochondrial functional status, including respiratory chain electron transport efficiency, membrane potential changes, and mitochondrial dynamics [55]. Thus, integrating transcriptomic data with metabolomics and mitochondrial functional profiling would provide a more comprehensive understanding of redox homeostasis. We also acknowledge that both HaCaT and TIGK used in this study are immortalized keratinocyte lines. However, previous reports have shown that these cell lines demonstrate good fidelity to their primary cell counterparts [13]. For example, Turabelidze et al. showed that primary oral keratinocytes migrate and proliferate faster than skin keratinocytes [12], phenotypes that were recapitulated in our cell lines. Additionally, future studies employing organoid models, three-dimensional co-culture systems, and single-cell sequencing approaches will be necessary to validate these differences in a more physiologically relevant context.

In summary, our study systematically delineates the differential responses of oral and skin keratinocytes to H_2_O_2_-induced oxidative stress. Consistent with the greater adaptability of oral tissues during wound healing and in response to injury, TIGK demonstrated enhanced tolerance and resilience compared with HaCaT, as evidenced by improved survival, reduced ROS accumulation, less SG formation, and superior migratory and proliferative recovery. These differences can be attributed to multiple layers of redox regulation: (1) under both basal and oxidative stress conditions, TIGK maintained higher expression of antioxidant enzymes such as SOD1, LPO, GSTM3, and TrxR, confirming its superior capacity to resist peroxidative damage under oxidative stress; (2) at baseline, TIGK expressed higher levels of key antioxidant genes, including *SOD1*, *TXN2*, *PRDX2*, and *PRDX6*, and further activated metal ion homeostasis pathways upon stress, conferring greater efficiency in ROS clearance and cytoprotection; (3) classical pro-oxidant genes, including NADPH oxidases and NOS, were expressed at lower basal levels in TIGK with a narrower induction range following stress and were more centered on metabolic homeostasis, thereby limiting excessive ROS generation at the source. Through these synergistic mechanisms, oral keratinocytes can more rapidly restore homeostasis and function under oxidative stress than HaCaT. Future work targeting these key pathways may provide promising strategies to enhance antioxidant defense and promote wound repair in skin.

## Data Availability

The RNA-seq datasets generated and analyzed during this study are available in the NCBI GEO database under accession number GSE308958. The R code used in this manuscript is available on GitHub: https://github.com/ChenHanMDPhD/TIGK-vs-HaCaT_Oxidative-Stress/blob/main/R%20Code%20for%20Manuscript (accessed on 23 December 2025). Other data are available at: https://doi.org/10.25417/uic.30534911.v1 (accessed on 23 December 2025).

## Supplementary Materials

The following supporting information can be downloaded at: https://www.mdpi.com/article/10.3390/cells15020097/s1. Supplementary Figure S1: Oral keratinocytes exhibit strong antioxidant capacity. Supplementary Figure S2: Enrichment analysis of differentially expressed genes (DEGs) upregulated in TIGK or HaCaT at baseline, showing Gene Ontology (GO) terms for biological processes (BP), molecular functions (MF), and cellular components (CC), as well as Reactome pathways. Supplementary Figure S3: Enrichment analysis of DEGs upregulated in TIGK or HaCaT after H_2_O_2_ treatment, showing GO terms for BP, MF, and CC, as well as Reactome pathways. Supplementary Figure S4: Enrichment of antioxidant genes (AOs) differentially up-regulated in TIGK or HaCaT at baseline, showing GO terms for BP, MF, and CC, as well as Reactome pathways. Supplementary Figure S5: Enrichment of AOs differentially up-regulated in TIGK or HaCaT after H_2_O_2_ treatment, showing GO terms for BP, MF, and CC, as well as Reactome pathways. Supplementary Figure S6: Enrichment of pro-oxidant genes (POs) differentially up-regulated in TIGK or HaCaT at baseline, showing GO terms for BP, MF, and CC, as well as Reactome pathways. Supplementary Figure S7: Enrichment of POs differentially up-regulated in TIGK or HaCaT after H_2_O_2_ treatment, showing GO terms for BP, MF, and CC, as well as Reactome pathways. Supplementary Table S1: Antioxidant gene set. Supplementary Table S2: Pro-oxidant gene set. Supplementary Table S3: Shared up AOs in TIGK and H_2_O_2_-treated HaCaT. Supplementary Table S4: Shared down POs in TIGK and H_2_O_2_-treated HaCaT.
