# Supplementary material for: Response of Oral and Skin Keratinocytes to Oxidative Stress
Source: Cells. 2026 Jan 6;15(2):97. doi: 10.3390/cells15020097 (PMC12839278; doi:10.3390/cells15020097)
Supplement: Supplementary file 1 [file cells-15-00097-s001.zip › Supplementary Figures.pptx]

## Slide 1
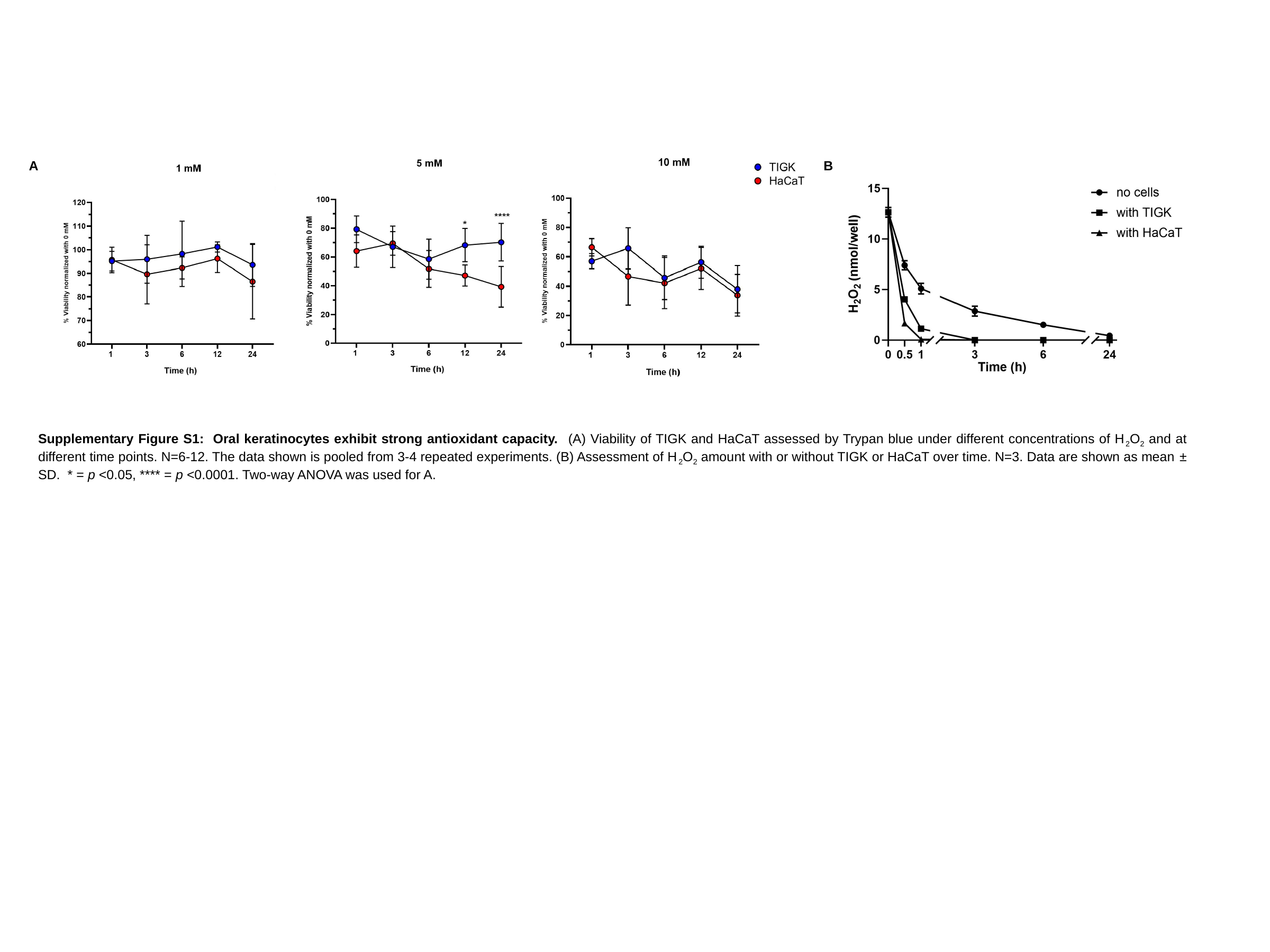

B
A
Supplementary Figure S1: Oral keratinocytes exhibit strong antioxidant capacity. (A) Viability of TIGK and HaCaT assessed by Trypan blue under different concentrations of H2O2 and at different time points. N=6-12. The data shown is pooled from 3-4 repeated experiments. (B) Assessment of H2O2 amount with or without TIGK or HaCaT over time. N=3. Data are shown as mean ± SD. * = p <0.05, **** = p <0.0001. Two-way ANOVA was used for A.

## Slide 2
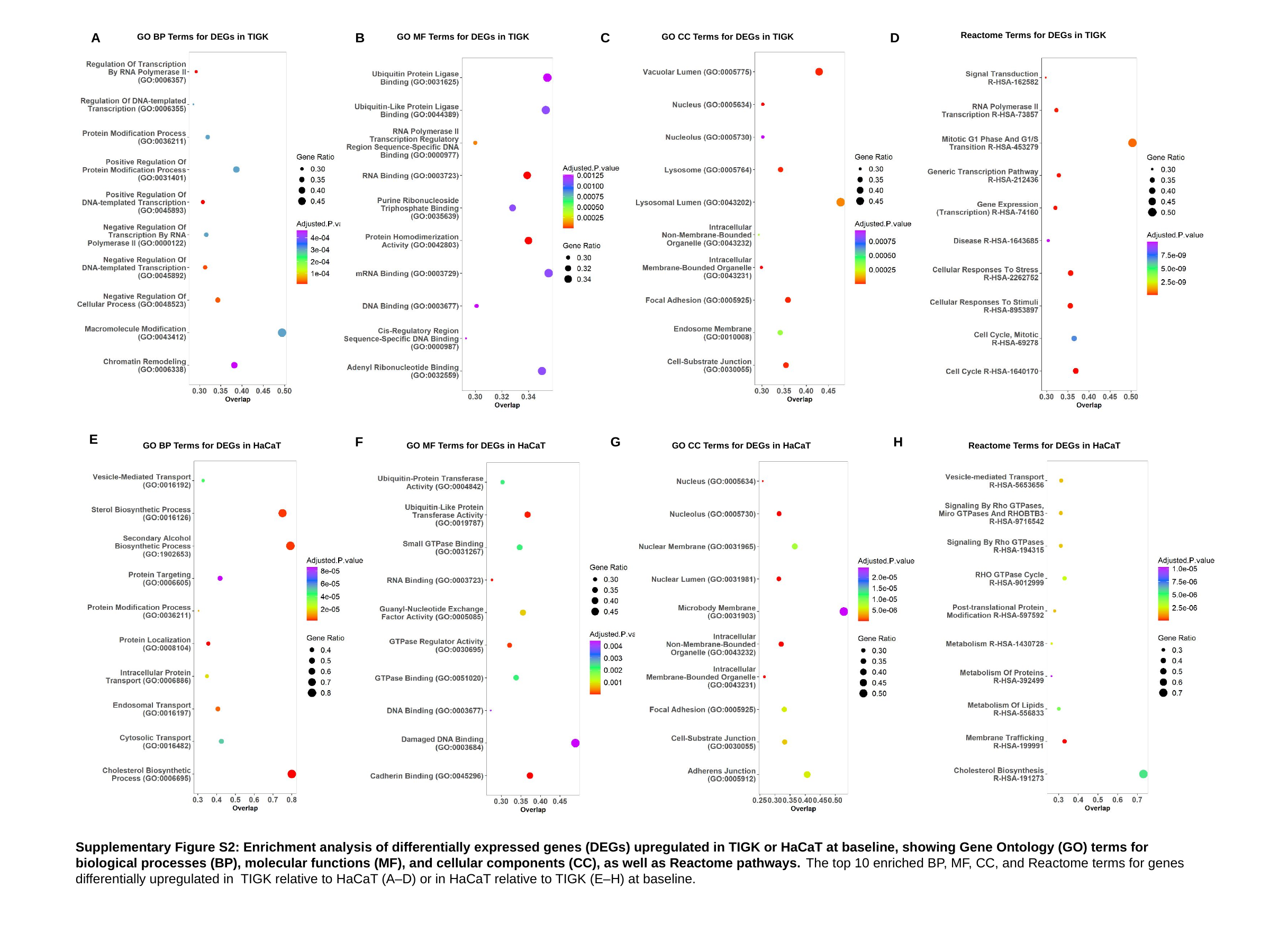

D
Reactome Terms for DEGs in TIGK
C
A
B
GO BP Terms for DEGs in TIGK
GO MF Terms for DEGs in TIGK
GO CC Terms for DEGs in TIGK
E
G
H
F
GO BP Terms for DEGs in HaCaT
GO MF Terms for DEGs in HaCaT
GO CC Terms for DEGs in HaCaT
Reactome Terms for DEGs in HaCaT
Supplementary Figure S2: Enrichment analysis of differentially expressed genes (DEGs) upregulated in TIGK or HaCaT at baseline, showing Gene Ontology (GO) terms for biological processes (BP), molecular functions (MF), and cellular components (CC), as well as Reactome pathways. The top 10 enriched BP, MF, CC, and Reactome terms for genes differentially upregulated in TIGK relative to HaCaT (A–D) or in HaCaT relative to TIGK (E–H) at baseline.

## Slide 3
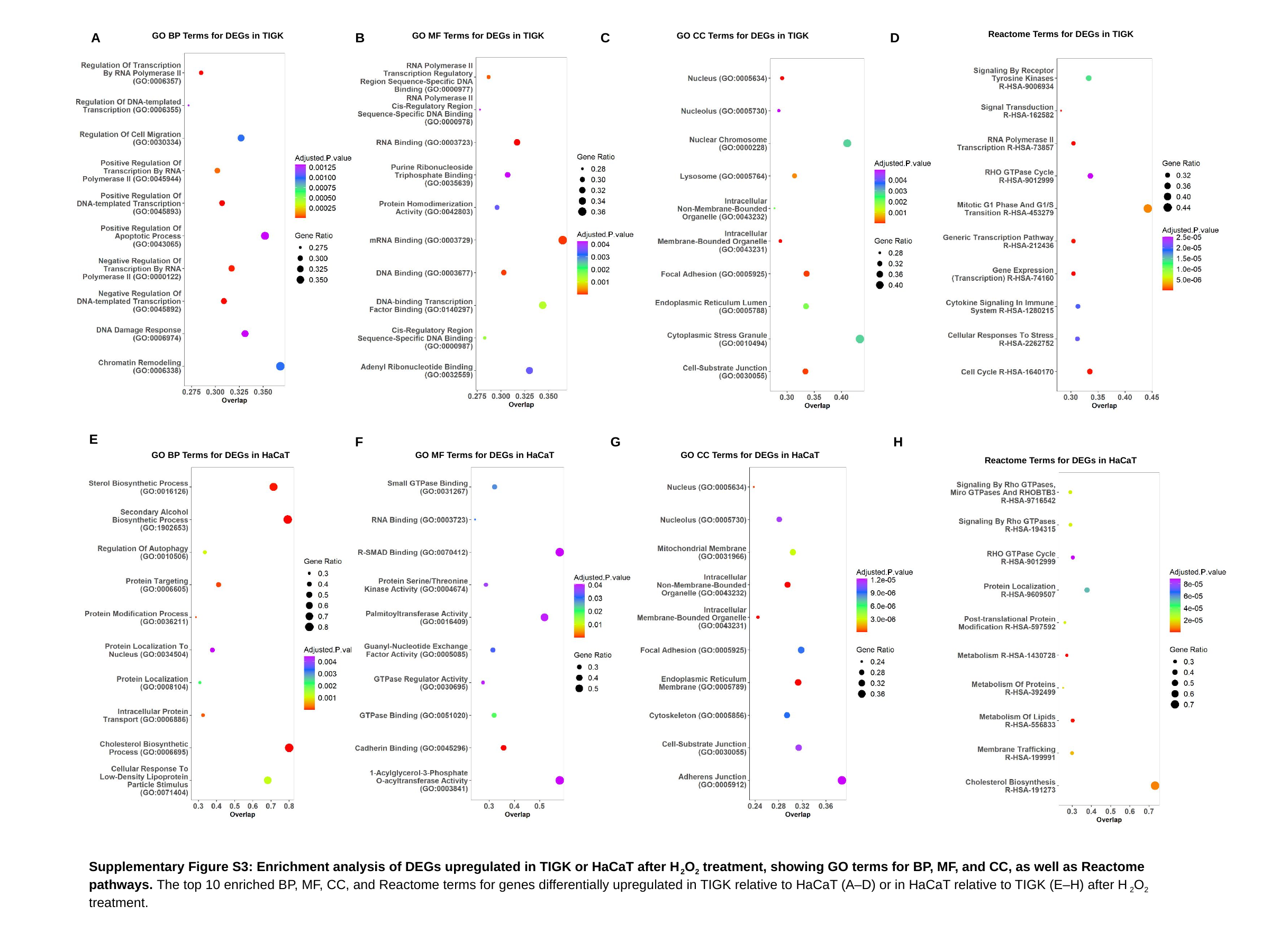

Reactome Terms for DEGs in TIGK
D
C
A
B
GO BP Terms for DEGs in TIGK
GO MF Terms for DEGs in TIGK
GO CC Terms for DEGs in TIGK
E
G
H
F
GO BP Terms for DEGs in HaCaT
GO MF Terms for DEGs in HaCaT
GO CC Terms for DEGs in HaCaT
Reactome Terms for DEGs in HaCaT
Supplementary Figure S3: Enrichment analysis of DEGs upregulated in TIGK or HaCaT after H2O2 treatment, showing GO terms for BP, MF, and CC, as well as Reactome pathways. The top 10 enriched BP, MF, CC, and Reactome terms for genes differentially upregulated in TIGK relative to HaCaT (A–D) or in HaCaT relative to TIGK (E–H) after H2O2 treatment.

## Slide 4
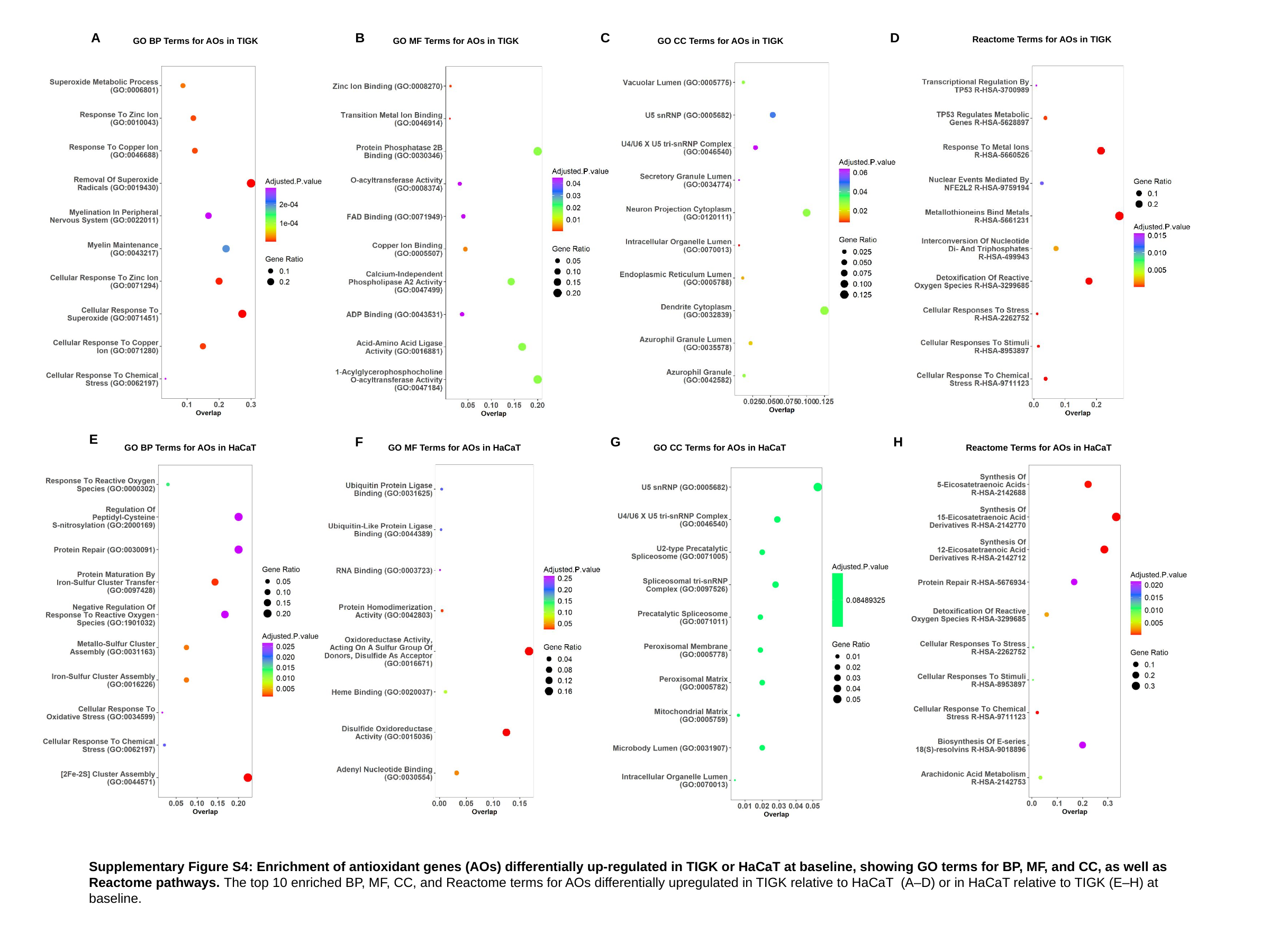

D
C
A
B
Reactome Terms for AOs in TIGK
GO BP Terms for AOs in TIGK
GO MF Terms for AOs in TIGK
GO CC Terms for AOs in TIGK
E
G
H
F
GO BP Terms for AOs in HaCaT
GO MF Terms for AOs in HaCaT
GO CC Terms for AOs in HaCaT
Reactome Terms for AOs in HaCaT
Supplementary Figure S4: Enrichment of antioxidant genes (AOs) differentially up-regulated in TIGK or HaCaT at baseline, showing GO terms for BP, MF, and CC, as well as Reactome pathways. The top 10 enriched BP, MF, CC, and Reactome terms for AOs differentially upregulated in TIGK relative to HaCaT (A–D) or in HaCaT relative to TIGK (E–H) at baseline.

## Slide 5
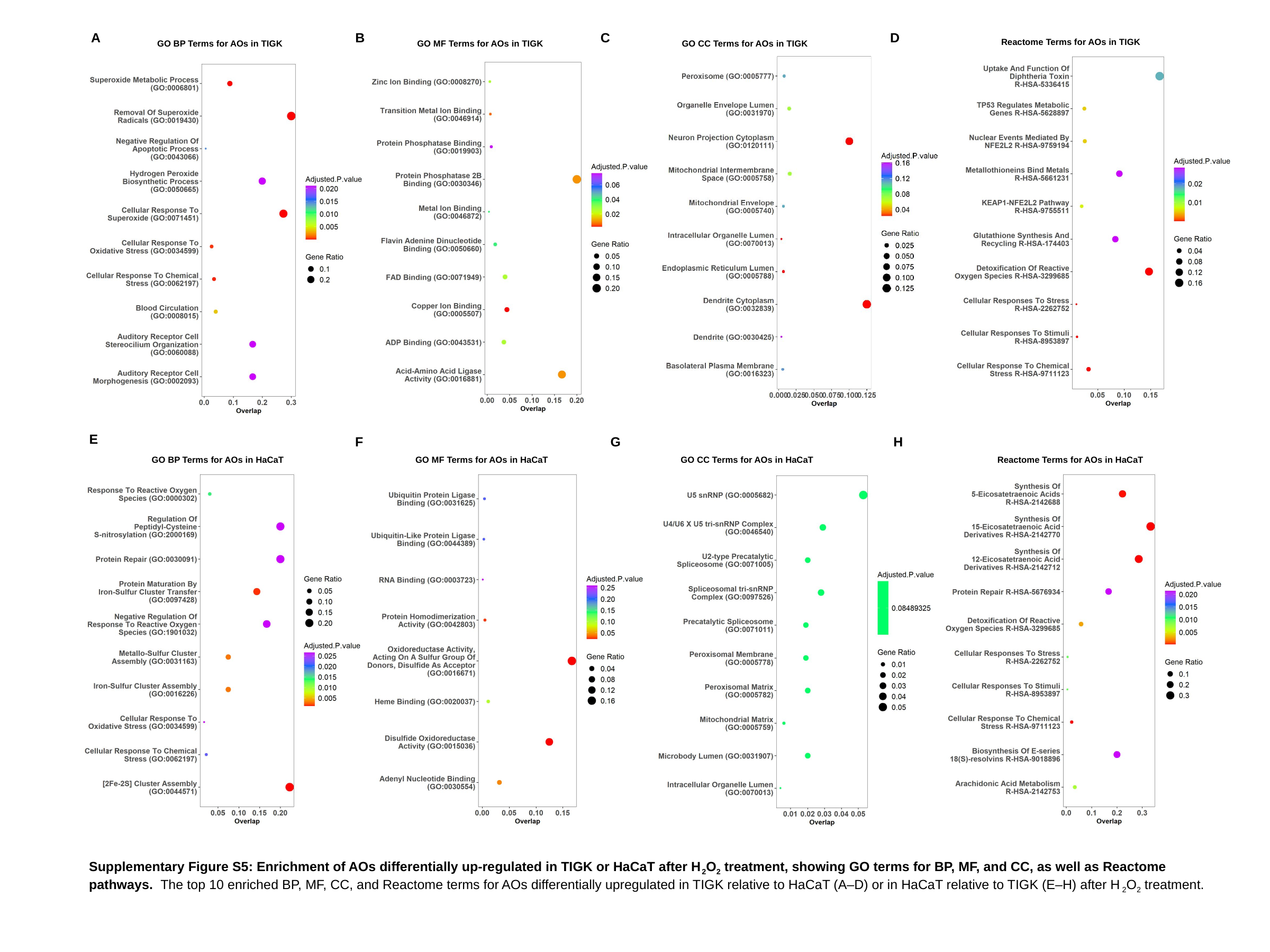

D
C
A
B
Reactome Terms for AOs in TIGK
GO BP Terms for AOs in TIGK
GO MF Terms for AOs in TIGK
GO CC Terms for AOs in TIGK
E
G
H
F
GO BP Terms for AOs in HaCaT
GO MF Terms for AOs in HaCaT
GO CC Terms for AOs in HaCaT
Reactome Terms for AOs in HaCaT
Supplementary Figure S5: Enrichment of AOs differentially up-regulated in TIGK or HaCaT after H2O2 treatment, showing GO terms for BP, MF, and CC, as well as Reactome pathways. The top 10 enriched BP, MF, CC, and Reactome terms for AOs differentially upregulated in TIGK relative to HaCaT (A–D) or in HaCaT relative to TIGK (E–H) after H2O2 treatment.

## Slide 6
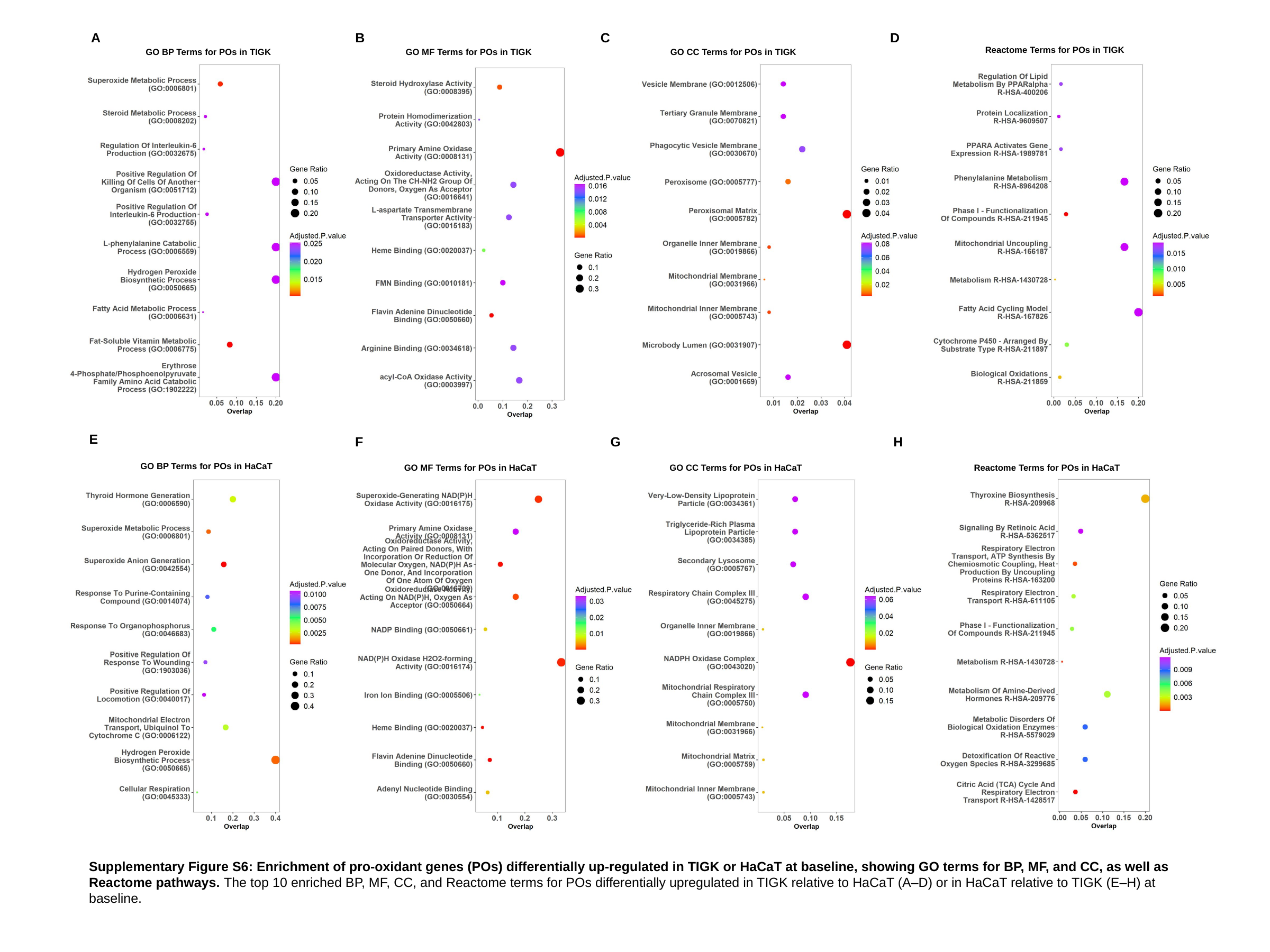

D
C
A
B
Reactome Terms for POs in TIGK
GO BP Terms for POs in TIGK
GO MF Terms for POs in TIGK
GO CC Terms for POs in TIGK
E
G
H
F
GO BP Terms for POs in HaCaT
GO MF Terms for POs in HaCaT
GO CC Terms for POs in HaCaT
Reactome Terms for POs in HaCaT
Supplementary Figure S6: Enrichment of pro-oxidant genes (POs) differentially up-regulated in TIGK or HaCaT at baseline, showing GO terms for BP, MF, and CC, as well as Reactome pathways. The top 10 enriched BP, MF, CC, and Reactome terms for POs differentially upregulated in TIGK relative to HaCaT (A–D) or in HaCaT relative to TIGK (E–H) at baseline.

## Slide 7
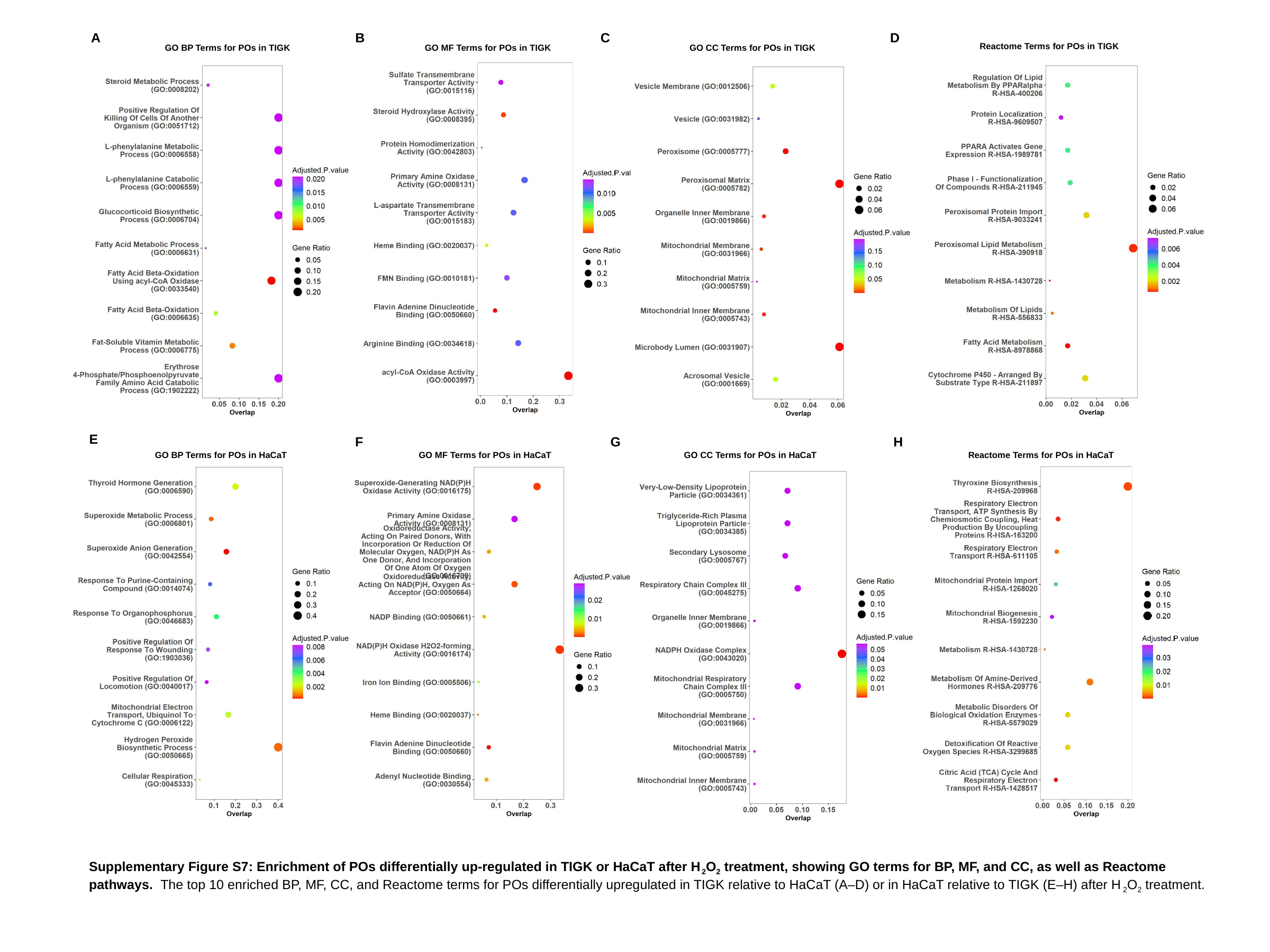

D
C
A
B
Reactome Terms for POs in TIGK
GO BP Terms for POs in TIGK
GO MF Terms for POs in TIGK
GO CC Terms for POs in TIGK
E
G
H
F
GO BP Terms for POs in HaCaT
GO MF Terms for POs in HaCaT
GO CC Terms for POs in HaCaT
Reactome Terms for POs in HaCaT
Supplementary Figure S7: Enrichment of POs differentially up-regulated in TIGK or HaCaT after H2O2 treatment, showing GO terms for BP, MF, and CC, as well as Reactome pathways. The top 10 enriched BP, MF, CC, and Reactome terms for POs differentially upregulated in TIGK relative to HaCaT (A–D) or in HaCaT relative to TIGK (E–H) after H2O2 treatment.
